# Structural recognition of tubulysin B derivatives by multidrug resistance efflux transporters in human cancer cells

**DOI:** 10.18632/oncotarget.18385

**Published:** 2017-06-06

**Authors:** Michal Stark, Yehuda G. Assaraf

**Affiliations:** ^1^ The Fred Wyszkowski Cancer Research Laboratory, Department of Biology, Technion-Israel Institute of Technology, Haifa 32000, Israel

**Keywords:** cancer, anti-microtubule agents, tubulysins, multidrug resistance, efflux transporters

## Abstract

Multidrug resistance (MDR) is a major hindrance to curative chemotherapy of various human malignancies. Hence, novel chemotherapeutics must be evaluated for their recognition by MDR efflux transporters. Herein we explored the cytotoxic activity of synthetic tubulysin B (Tub-B, EC1009) derivatives (Tub-B-hydrazide/EC0347 and Tub-B bis-ether/EC1820), and their recognition by the MDR efflux transporters P-glycoprotein 1 (P-gp), multidrug resistance-associated protein 1 (MRP1) and breast cancer resistance protein (BCRP). Originally isolated from *Myxobacteria*, tubulysins exhibited potent cytotoxic activity via microtubule depolymerization, and evaded recognition by these MDR efflux pumps. We show that subtle modifications in the natural Tub-B structure enhance its cytotoxicity and drug efflux efficiency. Whereas increasing the lipophilicity of Tub-B drugs enhanced their diffusion into the cell and consequently decreased the IC_50_ values (≥ 0.27 nM), increasing drug polarity enhanced their recognition by P-gp (>200-fold resistance in P-gp-overexpressing cells). Furthermore, restricting drug exposure time to the clinically relevant 4 h pulse, markedly enhanced efflux by P-gp, resulting in a 1000-fold increased resistance, which was further enhanced upon increased P-gp levels (i.e. an additional 3-fold increase in P-gp levels resulted in >6,000-fold resistance). The unique ability of EC1009 to evade recognition by MDR efflux pumps warrants drug development of tubulysin B derivatives as potent antitumor agents which overcome MDR in cancer.

## INTRODUCTION

In the ongoing quest for novel antitumor agents, researchers often turn to the natural pharmacopeia as a reliable source of multiple cytotoxic agents. In 2000, Reichenbach and his colleagues discovered four peptide-like compounds from strains of the solid bacteria *Myxobacteria*, containing unique amino acids; these natural products displayed potent cytostatic antitumor activity [1]. As these hydrophobic peptides exerted their cytotoxic activity via binding to tubulin cytoskeleton, they were named tubulysins; these include tubulysin A, B, D and E [1]. Following this discovery, multiple tubulysins were further isolated and characterized, i.e. tubulysins F-I [2] through Z [3]. The structure of tubulysins is distantly related to the marine slug alkaloids dolastatins, and their mode of action resembles that of the *Vinca* alkaloids vinblastine and vincristine as well as colchicine and combretastatins, i.e. binding to β-tubulin in the α-β heterodimeric form, hence inducing destabilization, inhibition of tubulin polymerization and microtubule (MT) depolymerization [1, 4]. This is opposed to MT-stabilizing agents including paclitaxel, docetaxel, epothilones, and discodermolide which bind to tubulin polymers and stabilize MT [5]. Nonetheless, both MT destabilizing and stabilizing agents alter the equilibrium between tubulin and MT, resulting in disruption of the mitotic spindle and cell death. Notably, tubulysins surpass the tumor cell growth inhibitory potential of vinblastine and the taxene paclitaxel by one to three orders of magnitude [2, 6].

Multidrug resistance (MDR) is a phenomenon in which cancer cells display resistance to a wide spectrum of anticancer drugs which are structurally and mechanistically distinct [7–10]. MDR, which can be classified either as intrinsic (i.e. emerging before chemotherapy) or acquired (provoked by chemotherapy), continues to be a dominant hindrance to curative cancer therapy [11–15]. Various mechanisms of cancer MDR have been described, albeit, active drug extrusion from cancer cells by ATP-driven efflux transporters remains a central mechanism of MDR [9, 15–18]. Enhanced drug efflux is predominantly mediated by ATP-dependent extrusion pumps of the ATP-binding cassette (ABC) superfamily, including P-glycoprotein 1 (P-gp, ABCB1/MDR1) [19–22], multidrug resistance-associated proteins (MRPs/ABCC) like MRP1 (ABCC1) [23, 24] as well as breast cancer resistance protein (BCRP/ABCG2) [25, 26]. These drug extrusion pumps couple the energy derived from ATP hydrolysis to the expulsion of a multitude of cytotoxic compounds which are structurally and mechanistically distinct. This potent drug efflux results in a marked decrease in the intracellular concentration of these antitumor agents, thereby conferring MDR to a wide array of chemotherapeutic agents [16, 27, 28]. Despite various approaches aimed at the overcoming of chemoresistance [12, 29–34], MDR continues to be one of the leading causes of chemotherapy failure and mortality.

**Figure 1:**
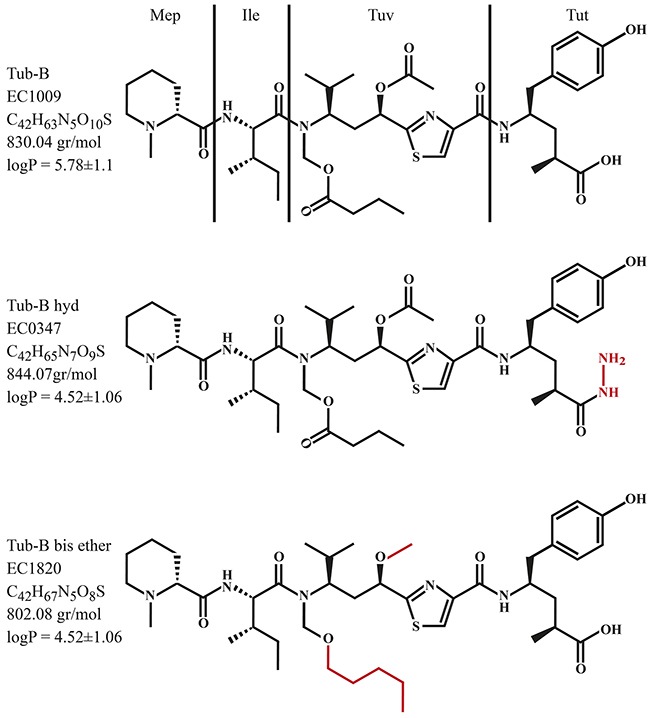
Chemical structures of Tub-B, EC1009; Tub-B-Hyd, EC0347; and Tub-B bis-ether, EC1820

Tubulysins exhibit relatively low substrate recognition by P-gp when compared to other anti-microtubule agents such as vinblastine, vincristine, vindesine, colchicine, paclitaxel and docetaxel [1, 6]. While tubulysins display characteristics of potent chemotherapeutic agents, they were purified from *Myxobacteria* in minute yields (<4 mg/liter of culture medium) [1, 2], hence hindering their commercial availability and clinical application. This challenge urged organic chemists to synthesize tubulysins [35–39]. Since tubulysins are extremely cytotoxic, several research groups have rationally designed tubulysin conjugates and nanoparticles to facilitate selective targeting of the drug to tumor cells, while preventing untoward toxicity to normal tissues [40–45]. For example, the synthesis of folic acid-tubulysin B conjugates was undertaken by Endocyte Inc. who developed novel synthetic tubulysin derivatives (i.e. tubulysin B-hydrazide, Tub-B-hyd; and tubulysin B methyl-ether; tubulysin B-ester) [42, 46]. Herein we studied the cytotoxic activity of Tub-B (EC1009) and its free analogues Tub-B-hyd (EC0347) and Tub-B bis-ether (EC1820), the structures of which are illustrated in Figure 1, as well as their recognition by the three dominant MDR efflux transporters P-gp, MRP1 and BCRP. We further explored the impact of the drug exposure time on the ability of P-gp to abolish the cytotoxic effect of these Tub-B analogues. We found that the original natural structure of Tub-B is best suited to evade substrate recognition and extrusion by these MDR efflux pumps. While the structural differences we explored enhanced the cytotoxic potential of these compounds, they markedly enhanced recognition by certain MDR efflux transporters. Moreover, the longer the exposure time to these fast-acting drugs, the smaller the effect of the MDR transporters on the cytotoxic outcome. These findings bear important implications for rational drug design and the overcoming of MDR in cancer.

## RESULTS

### Disruption of microtubules by synthetic Tub-B derivatives

**Figure 2:**
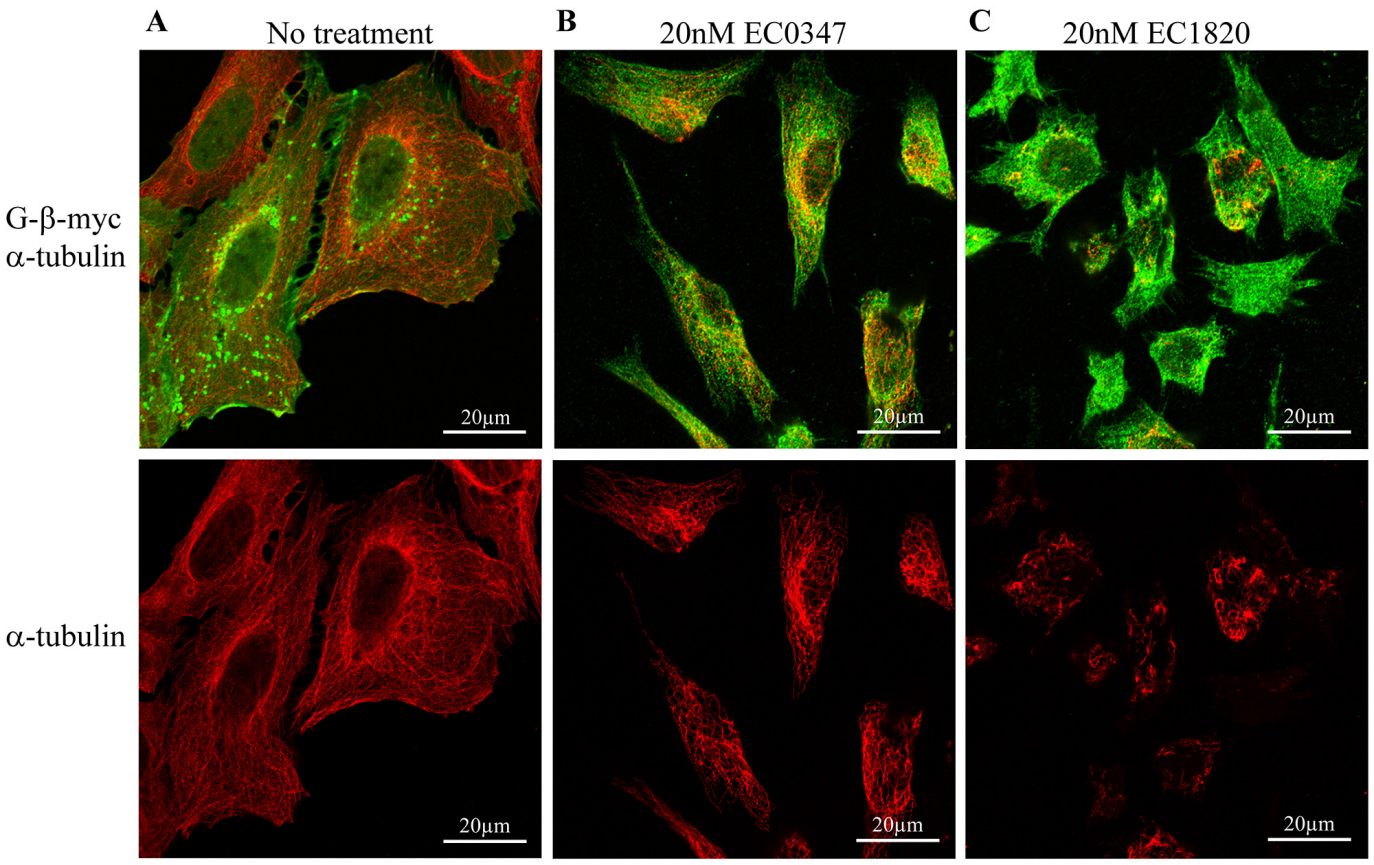
EC0347 and EC1820 disrupt the cellular microtubule network upon 4h drug exposure 2008/WT cells where transfected with a G-β-myc expression vector and subjected to a 4h pulse treatment with 20nM EC0347 or EC1820. Immunofluorescence microscopy was performed, as detailed under Materials and Methods, using drug-free control cells **(A)**, EC0347 **(B)**, and EC1820 **(C)** treated cells to evaluate the status of the microtubule network (red fluorescence), and G-β-myc (green fluorescence) as a plasma membrane marker.

Original studies by Reichenbach and colleagues have shown that a 4 hr pulse exposure of tumor cells to tubulysin A resulted in the disruption of cellular MT [1, 4], whereas the more active tubulysin D induced multipolar spindles [4]. Hence, to confirm that our tubulysin B derivatives also block microtubule polymerization in our tumor cell system, we used immunofluorescence microscopy with a β-tubulin-specific monoclonal antibody (Figure 2). Expectedly, 4 hr pulse treatment of human ovarian carcinoma 2008/WT cells with 20 nM of the Tub B derivatives EC0347 and EC1820 resulted in disruption of cytoskeletal MT (Figure 2). While treatment with EC0347 yielded a less pronounced microtubule network (compare Figure 2B with 2A, red fluorescence), EC1820 completely abolished any recognizable tubulin structure (compare Figure 2C with 2A, red fluorescence). The myc-tagged plasma membrane inhabitant G-β protein was used to trace the plasma membrane (compare Figure 2B and 2C with 2A, green fluorescence). Consistent with the original studies of Reichenbach and colleagues, these findings confirm that the Tub B derivatives EC0347 and EC1820 disrupt MT assembly at nanomolar drug concentrations upon clinically relevant 4 h pulse exposure.

### Tub-B-Hyd/EC0347 is a good substrate of P-gp

**Figure 3:**
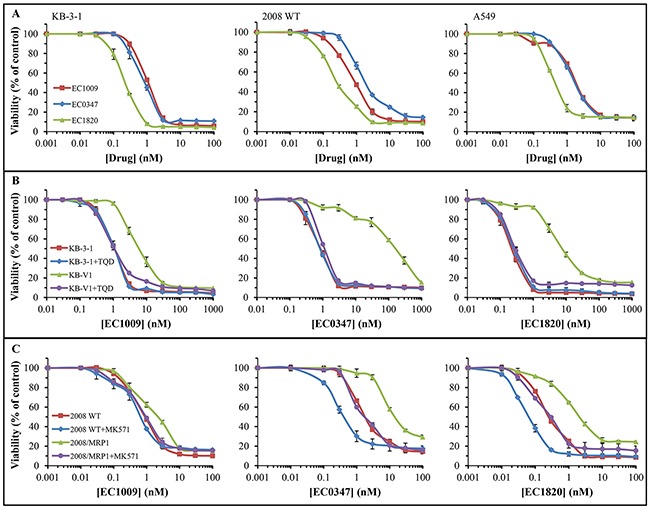
Cytotoxicity of Tub-B and its derivatives after 48h drug exposure Growth inhibition assays were undertaken with EC1009, EC0347 and EC1820, using the XTT cell proliferation kit. **(A)** Evaluation of the compounds' cytotoxicity in the three indicated parental cell lines. Differences between the curves at the IC_50_ concentration had *P-*values between 0.049 to 4×10^−5^. **(B)** Evaluation of the compounds' cytotoxicity in parental KB-3-1 cells and their P-gp overexpressing subline, KB-V1, in the absence or presence of the potent P-gp transport inhibitor Tariquidar (TQD). The differences displayed between the curves of KB-V1 cells (triangles) vs. all the others at the IC_50_ concentration had *P*-values ≤0.0065. **(C)** Evaluation of the compounds' cytotoxicity in parental 2008/WT cells and their MRP1-overexpressing subline, 2008/MRP1, in the absence or presence of the potent MRP1 transport inhibitor MK571. Results were normalized to the drug-free control for each treatment and are the means of at least three independent experiments performed in triplicates ±S.D. All the differences between the killing curves at the IC_50_ concentration had *P*-values between 0.037 to 2×10^−5^ excluding the curves of 2008/MRP1+MK571 vs. 2008/WT for EC1009 and EC0347 which were insignificant.

Based on this anti-microtubule activity, we next explored the cytotoxic potential of these novel Tub-B analogues; towards this end, we employed a small-scale model of the screening methodology of the NCI-60, which has been used by the Developmental Therapeutics Program (DTP) of the National Cancer Institute (NCI) to screen >100,000 chemical compounds since 1990. We thus used three established human MDR tumor cell lines overexpressing the dominant MDR efflux transporters P-gp, MRP1 and BCRP (i.e. KB-V1, 2008/MRP1 and A549/K1.5, respectively), their drug sensitive parental counterparts, as well as their specific MDR transport inhibitors TQD [47], MK571 [48] and Ko143 [49]. Cells were exposed to the various cytotoxic compounds for 48h in the presence or absence of these specific MDR efflux transporter inhibitors and cell viability was determined. Figure 3 depicts the killing curves obtained with each Tub-B compound and the IC_50_ values are summarized in Table 1. EC1820 was the most cytotoxic derivative in all three parental tumor cell lines 2008/WT, KB-3-1 and A549 (IC_50_ values 0.27±0.02, 0.28±0.07 and 0.47±0.04 nM, respectively), with 3-4-fold lower IC_50_ values compared to the parent drug and the other Tub-B derivative (Figure 3A, Table 1). EC1009 and EC0347 exhibited comparable IC_50_ values, except for parental 2008/WT cells. A549/K1.5 cells with BCRP overexpression [27] displayed only a minor cross-resistance towards these compounds (i.e. 27-38% increase in the IC_50_ values), when compared to their parental counterparts. Moreover, this minor cross-resistance was reversed by the specific BCRP transport inhibitor Ko143 only with the parent drug EC1009 (Table 1).

**Table 1:** Summary of results of growth inhibition assays upon drug exposure for 48h

Cell line	Tubulysin B EC1009			Tubulysin B hydrazide EC0347			Tubulysin B bis ether EC1820		
	IC_50_, nM	S.D.	Fold resistance	IC_50_, nM	S.D.	Fold resistance	IC_50_, nM	S.D.	Fold resistance
**A549**	1.96	0.01	1.00	1.83	0.04	1.00	0.47	0.04	1.00
**A549+Ko143**	2.11	0.06	1.07	1.91	0.13	1.04^a^	0.37	0.01	0.78
**A549/K1.5**	2.49	0.12	1.27	2.33	0.18	1.27	0.64	0.02	1.38
**A549/K1.5+Ko143**	2.00	0.02	1.02^a^	2.26	0.15	1.24	0.69	0.00	1.48
**KB-3-1**	1.08	0.04	1.00	0.85	0.09	1.00	0.28	0.07	1.00
**KB-3-1+TQD**	1.08	0.11	1.00^a^	0.84	0.05	0.99^a^	0.27	0.05	0.98^a^
**KB-V1**	6.55	1.06	6.06	177.05	23.00	209.11	8.09	0.82	29.24
**KB-V1+TQD**	1.16	0.07	1.07^a^	1.11	0.14	1.31	0.28	0.02	1.01^a^
**2008 WT**	0.92	0.05	1.00	1.94	0.11	1.00	0.27	0.02	1.00
**2008 WT+MK571**	0.70	0.06	0.76	0.45	0.06	0.23	0.07	0.01	0.26
**2008/MRP1**	2.33	0.09	2.54	16.59	0.43	8.57	2.35	0.03	8.85
**2008/MRP1+MK571**	0.97	0.03	1.05^a^	1.97	0.16	1.01^a^	0.30	0.01	1.11

All differences shown as fold of resistance have a *P*-value<0.05 excluding those indicated by an ^a^.

MRP1 displayed a higher degree of drug substrate recognition than BCRP, leading to a 2.5-8.8-fold increase in the IC_50_ values in the MRP1-overexpressing ovarian cancer cell line 2008/MRP1 [50]; co-treatment with the potent MRP1 transport inhibitor MK571 fully restored cellular sensitivity to these drugs, indicating that this drug resistance was solely mediated by MRP1. Furthermore, parental 2008/WT cells displayed a 4-fold decrease in the IC_50_ values towards the synthetic analogues in the presence of the MRP1 inhibitor (Table 1, *P*≤0.031). The highest cross-resistance factor was found in P-gp-overexpressing KB-V1 cells [51]. While EC1009 was the least recognized as an MDR transport substrate, with a modest 6-fold increase in drug resistance (*P*=0.0065), EC0347 was efficiently extruded by P-gp, hence leading to an increase as high as 209-fold in the IC_50_ value in KB-V1 cells (i.e. IC_50_ values of 177±23 vs. 0.85±0.09 nM, *P*=0.0028). Thus, the parent drug EC1009 was the least recognized as a transport substrate by the three MDR efflux transporters.

### Differential recognition of drugs by P-gp is most evident in clinically relevant drug exposure times

While the NCI-60 cytotoxicity screening assay is performed using a 48h drug exposure time, the actual clinical *i.v*. infusion time of tubulysin-cognate antitumor agents such as vinblastine and paclitaxel is 1 min and 3h, respectively [52]. In addition, the serum concentration of these drugs following *i.v*. administration rapidly decreases, with a 10-fold fall within several minutes to hours [53, 54]. Furthermore, we have previously demonstrated a time-dependent resistance to antifolates through the activity of the MDR efflux exporters MRP1 and BCRP [55, 56]. Taking these factors into consideration together with our immunofluorescence results, we undertook the same experiments with a much shorter tubulysin drug exposure time, i.e. a 4h pulse exposure, and evaluated its impact on P-gp-dependent Tub-B derivative drug resistance. Since parental KB-3-1 cells are devoid of detectable P-gp levels (Figure 5) [51, 57] and as TQD had no effect on the IC_50_ values in KB-3-1 cells during the 48h experiments, it was redundant to repeat TQD supplementation to these cells.

**Figure 4:**
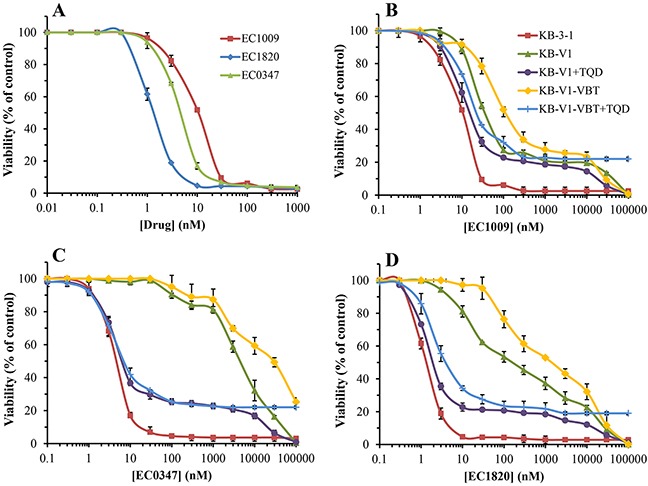
Cytotoxicity of Tub-B and its derivatives after 4h drug exposure **(A)** Growth inhibition was assessed after 4h drug exposure using increasing concentrations of EC1009, EC0347 and EC1820 in parental KB-3-1 cells. Differences between the IC_50_ values of all three drugs had *P*-values ≤0.0035. Evaluation of the cytotoxicity of EC1009 **(B)**, EC0347 **(C)** and EC1820 **(D)** in parental KB-3-1 cells, their P-gp-overexpressing subline KB-V1, and the newly vinblastine-selected KB-V1-VBT cells in the absence or presence of the potent P-gp transport inhibitor Tariquidar (TQD). All differences between the killing curves at the IC_50_ concentration had *P*-values between 0.043 to 4×10^−7^, excluding KB-V1+TQD (circles) vs. KB-V1-VBT+TQD (plus) which was insignificant for EC0347. Results were normalized to the drug-free control for each treatment and are the means of at least three independent experiments performed in triplicates ±S.D.

Limiting the drug exposure time to 4h had two prominent impacts: a) the IC_50_ values of the three Tub-B compounds increased by 5-10 fold, further expanding the difference in the cytotoxicity between the original parent drug EC1009 and its derivative EC1820 to 7.7-fold (vs. 3.9-fold upon 48h exposure, Figure 4A and Table 2). b) P-gp-dependent resistance towards the synthetic Tub-B analogues increased dramatically, i.e. 136-fold (*P*=0.0091) and 1072-fold (*P*=0.0001) for EC1820 and EC0347, respectively (Table 2 and Figure 4), while the resistance to the parent drug EC1009 was slightly decreased by 1.6-fold (i.e. 6-fold resistance at 48h vs. 3.7-fold resistance at 4h).

**Table 2:** Summary of results of growth inhibition assays upon drug exposure for 4h

Cell line	Tubulysin B EC1009			Tubulysin B hydrazide EC0347			Tubulysin B bis ether EC1820		
	IC_50_, nM	S.D.	Fold resistance	IC_50_, nM	S.D.	Fold resistance	IC_50_, nM	S.D.	Fold resistance
KB-3-1	10.42	0.11	1.00	4.31	0.38	1.00	1.35	0.06	1.00
KB-V1	38.67	2.58	3.71	4,616.0	114.00	1,072.2	183.83	43.38	136.17
KB-V1+TQD	16.67	1.97	1.60	6.99	0.41	1.62	2.06	0.08	1.52
KB-V1-VBT	134.21	0.44	12.89	26,327.0	212.85	6,115.5	1,595.2	140.30	1,181.6
KB-V1-VBT+TQD	24.21	0.19	2.32	7.69	1.44	1.79	4.27	1.81	3.16

All differences shown as fold of resistance have a *P*-value ≤0.026.

**Figure 5:**
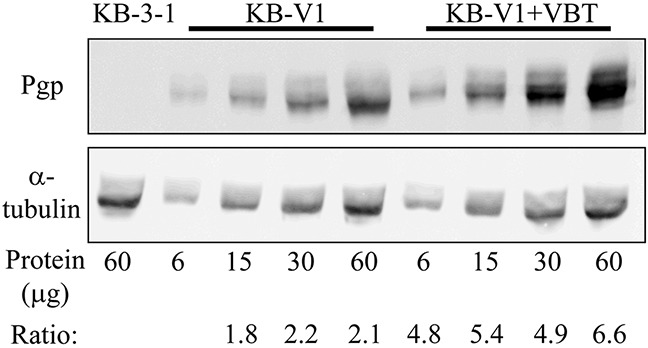
Western blot analysis of cellular P-gp expression Membrane proteins were extracted from parental KB-3-1 cells, their P-gp-overexpressing subline KB-V1, and the newly vinblastine-selected KB-V1-VBT cells, and the specified protein amounts were subjected to Western blot analysis using a P-gp-specific monoclonal antibody **(A)**. The membrane was stripped off and reacted with an α-tubulin antibody to confirm actual equal loading **(B)**. Quantification of the protein bands was performed using the EZ-Quant software, and the intensity ratio of P-gp/tubulin is indicated.

We next explored the impact of a further increase in cellular P-gp levels on the extent of drug resistance towards the various Tub-B derivatives. Towards this end, KB-V1 cells were further exposed for two weeks to 250 ng/ml vinblastine, the original selecting agent used for the establishment of the KB-V1 cell line, thus positively selecting for the highest P-gp-overexpressing cells. Western blot analysis revealed that these vinblastine-selected KB-V1 cells, i.e. KB-V1-VBT, acquired a 3-fold increase in P-gp levels relative to KB-V1 cells that were not further exposed to vinblastine (Figure 5). These newly selected cells were now assessed for their Tub B-derivative sensitivity using cytotoxicity assays. Remarkably, the 3-fold further increase in cellular P-gp levels markedly increased the IC_50_ values of the synthetic compounds when compared to the original KB-V1 cells; 1,595±140 nM *vs*. 183±43 nM (i.e. 8.7-fold, *P*=0.0019) for EC1820, as well as 26.3±0.2 μM vs. 4.6±0.11 μM (i.e. 5.7-fold, *P*=4×10^−7^) for EC0347, resulting in a dramatic 1,180-fold (*P*=0.00064) and 6,110-fold (*P*=5×10^−6^) resistance, respectively. Hence, the 3-fold increase in cellular P-gp levels resulted in 8.7-fold and 5.7-fold increased resistance to EC1820 and EC0347, respectively. In contrast, consistent with our previous results, the IC_50_ value of the parent EC1009 compound was only slightly increased by 3.5-fold (Figure 4 and Table 2, *P*=5×10^−7^). Table 3 summarizes the recognition and efflux capacity of the tubulysin B derivatives by the different MDR efflux transporters; hence, whereas BCRP failed to recognize any of these compounds, MRP1 displayed a poor drug extrusion of EC1009 and a moderate drug efflux of EC0347 and EC1820. In contrast, whereas the parent drug EC1009 was a poor P-gp substrate, EC0347 and EC1820 were *bona fide* P-gp efflux substrates as evidenced by their efficient extrusion by this efflux pump.

**Table 3:** Recognition and expulsion of tubulysin B derivatives by MDR efflux transporters

	EC1009	EC0347	EC1820
**BCRP**	-	-	-
**MRP1**	+	++	++
**P-gp**	+	+++++	+++

## DISCUSSION

Here we studied the impact of structural modifications in Tub-B on the cytotoxic activity as well as recognition by the dominant MDR efflux transporters P-gp, MRP1 and BCRP. We also assessed the role of subtle increases in cellular P-gp levels on the MDR to these tubulysin B derivatives. In an attempt to mimic the clinical drug treatment with anti-microtubule agents, we finally studied the impact of the drug exposure time on the efficiency of the drug extrusion capacity of P-gp and consequent drug resistance.

In the ever expanding field of MT inhibitors currently used as chemotherapeutic agents [5, 58, 59], tubulysins attract much scientific interest. Various tubulysin analogues have been recently developed in an attempt to introduce novel small molecules which are relatively easy to synthesize, and to better understand the role of each residue of the molecule in MT binding and cytotoxic activity [60–66]. However, the vast majority of these MT-targeted drugs lost the potency of the original parent tubulysins. In the current study, we studied two Tub-B derivatives which were found to be as potent as the parent Tub-B drug, namely Tub-B-hyd (EC0347), or even exceeded the activity of the parent drug by a factor of 3.5-7.7 in the case of Tub-B bis-ether (EC1820). Tubulysins were previously found to be highly cytotoxic, with a high correlation (R^2^ = 0.95) between their lipophilicity and cytotoxic activity [2], as well as their ability to successfully evade recognition by the MDR efflux transporter, P-gp [1]. We consistently show here that whereas the parent EC1009 was a poor P-gp efflux substrate, its hydrazide derivative EC0347 was an excellent P-gp transport substrate; specifically, EC0347 was efficiently extruded by MDR KB-V1 cells with P-gp overexpression, hence achieving a very high level of MDR that could be fully reversed by TQD, a potent P-gp transport inhibitor. It should be emphasized that the sole chemical difference between the parent EC1009 drug and its hydrazide derivative EC0347 is the mere introduction of a hydrazide group in the tubutyrosine residue (Tut, Figure 1). The terminal amino group in this hydrazide residue is positively charged under physiological conditions (pH of 7.3). Hence, unlike its parent EC1009 drug, EC0347 is a hydrophobic cationic drug at pH 7.3. In this respect, it is well established that multiple P-gp transport substrates with distinct structures and modes of action, as well as transport inhibitors (known as MDR chemosensitizers), are lipophilic cationic compounds [67–75]. Furthermore, elimination of a basic center from the *bona fide* P-gp substrate doxorubicin overcame P-gp-dependent MDR, as this anthracycline was no longer recognized by P-gp as a transport substrate [76]. Remarkably, this apparently strong requirement for a cationic charge in lipophilic substrates of P-gp has been also identified in bacterial MDR transporters; it has been demonstrated that a single membrane-embedded negative residue in MdfA, the bacterial MDR efflux ancestor of P-gp, is absolutely required for the binding of a basic residue in lipophilic toxic compounds such as ethidium bromide and benzalkonium [77].

**Figure 6:**
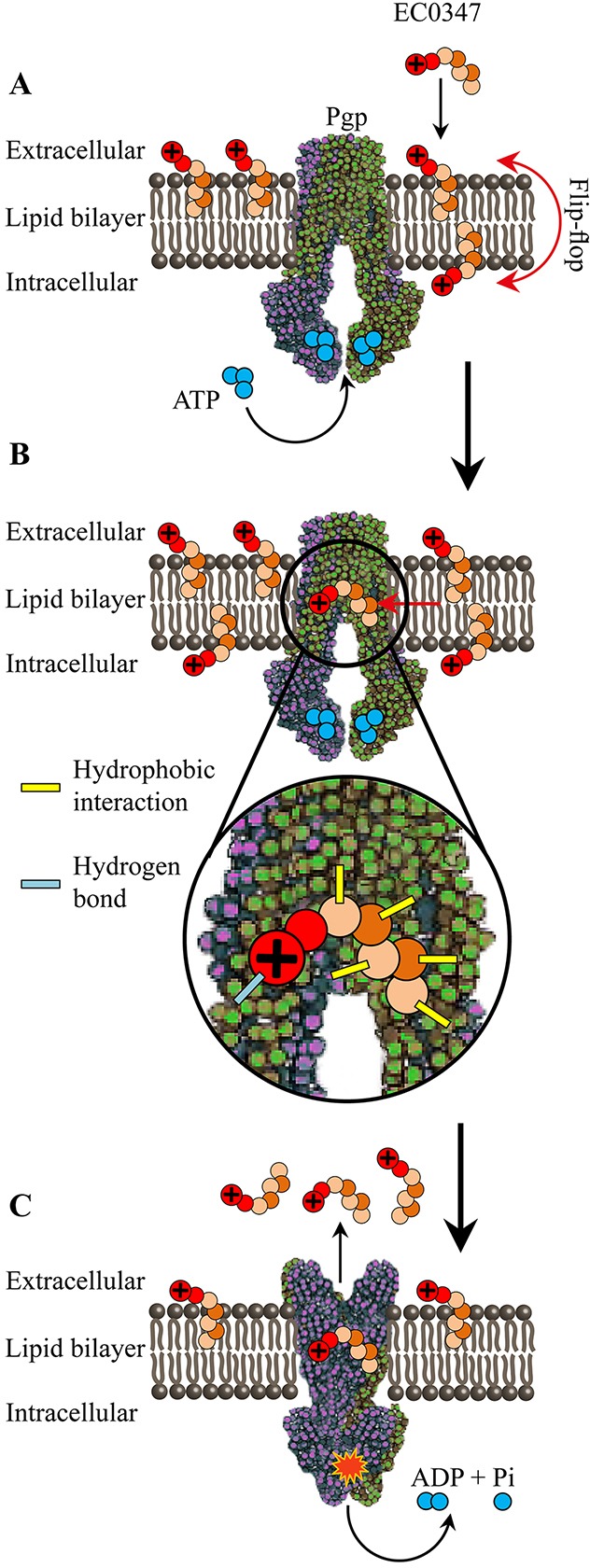
Proposed schematic model for the intercalation of EC0347 into the plasma membrane, its recognition in the lipid-bilayer by P-gp and its ATP-dependent extrusion by this MDR efflux pump **(A)** Intercalation of the cationic hydrophobic EC0347 into the plasma membrane with the positively charged residue protruding to the extracellular milieu or into the cytoplasmic face, following a putative slow flip-flop to the inner membrane leaflet. **(B)** Recognition of EC0347 by P-gp in the lipid core of the phospholipid bilayer via hydrophobic interactions and hydrogen bond formation. **(C)** Putative ATP-driven, P-gp-dependent flip-flop of EC0347 into the outer membrane leaflet and expulsion into the extracellular milieu.

It has been previously shown that P-gp is an ATP-driven unidirectional phospholipid flippase [17], transporting phospholipids from the inner to the outer leaflet of the lipid bilayer [78]. In this respect, we as well as others have previously shown that uncharged hydrophobic anticancer drugs (such as EC1009) traverse the plasma membrane very rapidly and exhibit an extremely short residence time in the lipid core of biomembranes. This membrane residence time is too short to allow for binding by P-gp and efficient drug extrusion [79]. In contrast, *bona fide* hydrophobic cationic P-gp transport substrates including EC0347 would undergo a relatively slow flip-flop in the plasma membrane (Figure 6), hence allowing sufficient time for P-gp to efficiently bind and expel them out of cells, thereby resulting in a high level of MDR [80–85]. Based on the crystal structure of the human P-gp [22], the large drug-binding site of P-gp (6,000 cubic Å) is rich in hydrophobic and aromatic amino acids but also contains polar amino acids. Hence, from a mechanistic perspective, we propose here that the lipophilic cation EC0347, which must undergo a slow flip-flop in the plasma membrane in order to traverse it, can be initially docked via hydrophobic interactions at the highly hydrophobic binding site of P-gp, followed by hydrogen bond formation between the polar residues in the drug-binding site of P-gp and the amino group of the hydrazide, thereby culminating in efficient drug expulsion after ATP hydrolysis (Figure 6).

In the current study we also explored minor modifications in the central unusual amino acid tubuvaline (Tuv, Figure 1), which is crucial for the compounds' hydrophobic core as well as the binding to, and destabilization of MT [86]. The elimination of two polar C=O double bonds in EC1820 increased the lipophilicity of the compound in comparison to the parent EC1009 drug (Log P=6.32±1.1 *vs*. 5.78±1.1). This markedly enhanced the anti-proliferative activity of the compound (IC_50_ values decreased by 3.4-4.2-fold in the different parental tumor cell lines after 48h drug exposure and as much as 7.7-fold after 4h pulse exposure); this presumably occurs by improving its diffusion rate across the plasma membrane. Moreover, this modification increased the recognition of EC1820 by P-gp (5-fold increased resistance of KB-V1 cells at 48h, and 36-fold enhanced resistance at 4h, compared to EC1009). Introduction of an alteration in the γ-amino acid homologue of tyrosine, tubutyrosine (Tut, Figure 1), which increases the polarity of the compound EC0347 (Log P=4.52±1.06) by replacing the hydroxyl group with an amide –NH-NH_2_, had little or no effect on the IC_50_ values. On the other hand, recognition by P-gp as an efflux substrate was highly enhanced by a factor of 35-fold upon 48h drug exposure and as much as 120-fold at 4h. As detailed above, we propose that the amino group of the hydrazide residue of EC0347 undergoes protonation at physiological pH, conferring an amphiphilic nature to the compound, thus markedly decreasing its diffusion across the plasma membrane which presumably proceeds via a flip-flop mechanism. As discussed above, since P-gp binds and expels its substrates during their diffusion through the lipid-core of the plasma membrane much before they reach the cytoplasm [20], the increased polarity of EC0347 and hence its presumed slower diffusion rate across the plasma membrane renders it a much better P-gp transport substrate than the parent drug EC1009.

While P-gp recognizes only lipophilic substrates, MRP1 transports both hydrophilic and hydrophobic compounds with diverse structures [87]. The parental drug EC1009 was inefficiently recognized and extruded by MRP1 with a 2.5-fold increase in the resistance of MRP1-overexpressing 2008/MRP1 cells, relative to parental 2008/WT cells. Recognition by MRP1 was increased following the structural alterations detailed above, resulting in an increase of ∼3.4-fold in the resistance towards EC0347 and EC1820 when compared to the parent drug EC1009. While EC1009 and EC0347 exhibited comparable IC_50_ values in both parental A549 and KB-3-1 cells, 2008/WT cells displayed a 2-fold increase in the IC_50_ value of EC0347 compared to EC1009. This could be explained by the fact that unlike parental A549 and KB-3-1 cells that do not express any detectable levels of MDR transporters [27, 57], 2008/WT cells express endogenous MRP1 [88]. This ubiquitous MRP1 expression resulted in cellular resistance to both EC0347 and EC1820, which was fully reversed by the MRP1 inhibitor MK571, as evidenced by the 4-fold hypersensitivity.

The spectrum of BCRP drug substrates is wide and diverse with the vast majority of BCRP transport substrates harboring a hydrophobic polyaromatic structure with the typical presence of residues which are capable of forming hydrogen bonds with polar amino acids in the binding site of BCRP [26, 89]. Given that tubulysins are predominantly linear peptides lacking a multi-aromatic ring structure, it is not surprising that they were not BCRP transport substrates.

The pre-clinical NCI-60 drug screening platform is widely used, although it is not clinically relevant for the extrusion of multiple anticancer drugs as most drugs are *i.v*. administered over several hours and not several days. We showed here that upon a short 4h drug exposure, which is more representative of the clinical bolus treatment with chemotherapeutic agents, P-gp overexpression had a markedly greater impact on drug efflux than the long-term drug exposure of 48h. The drug resistance of KB-V1 cells towards the modified Tub-B compounds substantially increased upon shortening the drug exposure time, achieving >1000-fold resistance to EC0347. The parent EC1009 drug which was the least recognized by P-gp, efficiently evaded this drug efflux pump during the 4h exposure period, thereby leading to a decrease in the resistance of KB-V1 cells (i.e. 3.7-fold *vs*. 6-fold resistance at 48h, compared to KB-3-1 cells). This finding shows that a short drug exposure time markedly expands the difference between P-gp transport substrates and non-substrates, and hence is more revealing in screening and evaluation of novel cytotoxic compounds. This conclusion was further corroborated when P-gp expression levels were boosted up by further exposing KB-V1 cells to vinblastine; KB-V1-VBT cells exhibited a remarkable resistance towards EC1820 (1,100-fold), and as high as 6,100-fold resistance to EC0347 when compared to the 12-fold resistance towards the parent EC1009 drug.

Taken collectively, these novel findings have important implications for rational drug design of the proper structural elements of tubulysin B derivatives necessary to evade and overcome ABC transporter-dependent MDR. Hence, unlike multiple naturally occurring cytotoxic alkaloids and other hydrophobic compounds which are typically recognized by P-gp and BCRP including anthracyclines, *Vinca* alkaloids, epipodophyllotoxins, actinomycin D, epothilones, dolastatins, gramicidin D, and camptothecins, the parent tubulysin B drug largely evaded drug efflux via MDR pumps [79, 80, 89]. This unique and unusual feature warrants further drug development of tubulysin B derivatives as potent antitumor agents which overcome MDR.

## MATERIALS AND METHODS

### Materials

Tubulysin B/EC1009 (PubChem CID: 12134545), Tubulysin B hydrazide/EC0347 (PubChem CID: 52948106) and Tubulysin B bis-ether/EC1820 were obtained from Endocyte Inc. (West Lafayette, IN, USA). Patent publication number EP2908818 A2. Ko143 (PubChem CID: 10322450) was from Santa Cruz Biotechnology (Dallas, Texas, USA), Tariquidar (TQD, PubChem CID: 148201) was from MedKoo Biosciences (Chapel Hill, NC, USA) and MK571 (PubChem CID: 16760569) was purchased from Sigma Aldrich (St. Louis, MO, USA).

### Cell culture

Human cervical carcinoma KB-3-1 cells and their P-gp-overexpressing KB-V1 subline were maintained in growth medium containing one part of DMEM and two parts of folate-free DMEM (Sigma Aldrich, St. Louis, MO, USA); DMEM was supplemented with 10% fetal bovine serum (FBS), whereas folate-free DMEM was supplemented with dialyzed FBS (Biological Industries, Beit HaEmek, Israel). These cell lines were originally obtained from Prof. Michael M. Gottesman in 1993 and were subsequently verified for their overexpression of P-gp and folate receptor α by Western blot analysis. Human A549 non-small cell lung cancer cells and their BCRP-overexpressing subline A549/K1.5 (established by Prof. A. Skladanowski and characterized by us [27]) as well as human ovarian carcinoma 2008/WT and their MRP1-overexpressing 2008/MRP1 cells (received from Prof. Piet Borst and verified for MRP1-overexpression by Western blot and flow cytometry analyses [88]) were maintained in RPMI-1640 medium (Gibco, Life Technologies, Grand Isle, NY) supplemented with 10% FBS. All these tumor cell lines were grown in the presence of 2 mM glutamine and 100 μg/ml penicillin and streptomycin (Biological Industries, Beit HaEmek, Israel) in a humid atmosphere of 5% CO_2_ at 37°C. To maximize P-gp overexpression, KB-V1 cells were further selected for two weeks in the presence of 0.25 μg/ml vinblastine (Sigma Aldrich, St. Louis, MO, USA), thereby yielding KB-V1-VBT cells.

### Growth inhibition assay

Cells were seeded in 96-well plates. After 48h, the growth medium was replaced by a fresh medium containing or lacking the appropriate MDR efflux transporter inhibitor (i.e. 400nM TQD, 400nM Ko143, or 50μM MK571), followed by immediate addition of increasing concentrations of EC1009, EC1820 or EC0347. After 4h or 48h, monolayer cells were washed twice with growth medium. For the 4h drug exposure experiments, cells were allowed to grow for an additional 44h before growth inhibition was determined using a colorimetric cell proliferation kit (XTT, Biological Industries, Beit HaEmek, Israel). Percent inhibition of cell growth was calculated relative to drug free controls. IC_50_ is the drug concentration exerting 50% cell death. Results presented were obtained from at least three independent experiments performed in triplicates. We used a one-tailed paired Student's *t*-test to examine the significance of the differences between the IC_50_ values obtained for each cell line and/or drug (N≥3). A difference was considered significant if the *P*-value obtained was <0.05.

### Western blot analysis

Total protein lysates were extracted and Western blot analysis was performed as previously described [88]. Following extraction, protein concentration was determined by the Bradford protein assay (Bio-Rad, Hercules, CA, USA). Proteins were resolved on 7% polyacrylamide gels and blotted onto a Protran BA83 cellulose nitrate membrane (Whatman, GE, Maidstone, UK), and reacted with an anti-P-gp monoclonal antibody (JSB-1, kindly provided by Dr. G. Scheffer, VU Medical Center, Amsterdam, The Netherlands). The membrane was then reacted with a horseradish peroxidase-conjugated goat anti-mouse secondary antibody (Jackson Immunoresearch Labs, West Grove, PA). The membrane was stripped off and finally reacted with an anti-α-tubulin antibody for evaluation of actual loading (Sigma Aldrich, St. Louis, MO, USA). Quantification of the protein bands was performed using the EZ-Quant software (EZ-Quant, Tel-Aviv, Israel).

### Immunofluorescence

2008/WT cells were seeded in 24-well plates on sterile glass coverslips and transfected with pcDNA3.1/Gβ1-myc-His expression vector (kindly provided by Prof. David Meiri, Dept. of Biology, Technion, Haifa, Israel) using linear polyethylenimine (PEI, MW 25,000) transfection reagent (Polysciences, Pennsylvania, USA) at a ratio of 3μg PEI : 1μg DNA. 20h after transfection, cells were incubated in the presence of either 20 nM EC0347 or 20nM EC1820 for 4h. Then, cells were washed with medium, fixed [4% formaldehyde in PBS, for 15min at room temperature (RT)] and permeabilized using 0.1% Triton X100 for 5 min. Blocking was performed with a blocking solution (20% skimmed milk in TBS) for 1hr at RT followed by co-incubation with primary anti-myc antibody (1:250, Abcam, Cambridge, UK) and anti-α-tubulin (1:500, Sigma-Aldrich, St. Louis, MO, USA) in 20% blocking solution for 1hr at RT. Coverslips were washed 3 times with PBS and co-incubated with Dylight 488-conjugated donkey anti-Rabbit IgG and Dylight 594-conjugated donkey anti-mouse secondary antibodies (Jackson ImmunoResearch, West Grove, PA, USA), for 1hr at RT. After three washes with PBS, coverslips were mounted onto glass slides using Fluoromount-G (Southern Biotechnology Associates) and examined using a confocal Zeiss LSM 710 microscope.
